# Rapid axially scanned and de-scanned line-scan confocal microscopy with a tunable acoustic gradient index of refraction lens for high-speed volumetric *in vivo* imaging

**DOI:** 10.1117/1.NPh.12.4.045013

**Published:** 2025-12-22

**Authors:** Khuong Duy Mac, Suhyeon Kim, Tien Nhat Nguyen, Christine Hwang, Minsung Kim, Rui Liu, Yan Liu, Joon Heon Kim, Young Ro Kim, Euiheon Chung, Hyuk-Sang Kwon

**Affiliations:** a Gwangju Institute of Science and Technology, Department of Biomedical Science and Engineering, Gwangju, Republic of Korea; b University of California at San Diego, Department of Physics, La Jolla, California, United States; c Indiana University, School of Optometry, Bloomington, Indiana, United States; d Gwangju Institute of Science and Technology, Advanced Photonics Research Institute, Gwangju, Republic of Korea; e Massachusetts General Hospital, Athinoula A. Martinos Center for Biomedical Imaging, Charlestown, Massachusetts, United States; f Harvard Medical School, Department of Radiology, Boston, Massachusetts, United States; g Gwangju Institute of Science and Technology, AI Graduate School, Gwangju, Republic of Korea; h Gwangju Institute of Science and Technology, Research Center for Photon Science Technology, Gwangju, Republic of Korea

**Keywords:** axially scanned and de-scanned, cerebral blood flow imaging, volumetric *in vivo* imaging, line-scan confocal microscopy, tunable acoustic gradient index of refraction lens

## Abstract

**Significance:**

Rapid acquisition of high-resolution volumetric images has been critical to effectively monitor dynamic biological processes *in vivo*, yet it faces tradeoffs between image resolution, penetration depth, and imaging speed. These limitations hinder the ability to study rapid neurophysiological events such as cerebrovascular dynamics and cellular activity, highlighting the need for advanced high-speed 3D imaging system.

**Aim:**

To address these challenges in volumetric imaging performances, we aimed to develop a high-speed volumetric imaging system capable of resolving fast biological dynamics with minimal compromise in spatial resolution or imaging depth.

**Approach:**

We devised a rapid axially scanned and de-scanned (RASAD) scheme by integrating a TAG lens (tunable acoustic gradient index of refraction lens) into a line-scan confocal microscope. The TAG lens enabled axial (depth) scanning frequency at 70 kHz, allowing 3D projection imaging at rates up to 200 Hz with a detection depth of 135  μm while minimally sacrificing the image quality (i.e., a lateral resolution of ∼2.6  μm).

**Results:**

We validated its performance through *in vitro* imaging of spontaneously contracting cardiomyocyte aggregates, capturing real-time calcium transients and synchronized contractions, and through *in vivo* imaging of the mouse cortical tissue, where volumetric acquisition over a $450\times 450\times 100\;{\mu m}^{3}$ region enabled quantification of blood flow velocities up to 3.64  mm/s across various vessel types.

**Conclusions:**

The RASAD system enables high-speed, high-resolution 3D imaging of dynamic biological processes, providing a valuable tool for advancing studies of neurophysiological mechanisms and biomedical applications.

## 1 Introduction

In studies of living organisms, fluorescence imaging introduced a highly efficient, noninvasive means for scrutinizing biological events and processes at the microscopic level with high spatiotemporal resolution.^1^^,^^2^ The method has been widely applied, especially in the investigation of biomedical mechanisms, by exposing structures and functions via multiscale visualization of biological and physiological factors such as blood vessel formation, blood flow, distributions of cellular populations, and related dynamic activities.^3^ Optical manipulations and methods, such as light-sheet^4^^,^^5^ and confocal microscopy,^6^^,^^7^ were implemented together to further increase the versatility of the fluorescence imaging by incorporating already available technologies. The advantage of attaining volumetric data in biomedical applications is apparent, particularly for assessing tissue areas with depth-dependent biological features. However, additions of volume acquisition capability typically come at the cost of sacrificing either imaging speed or resolution, often resulting in a significant loss of spatiotemporal information.

In this regard, both lateral and axial scan speeds should be optimized to enhance 3D imaging efficiency with an adequate signal-to-noise ratio (SNR). Researchers have devised a few novel instrumentations and methods to improve the lateral scan speed. For example, a polygonal mirror, a deformable mirror, and an acousto-optic deflector^8^ have been implemented to increase the imaging speed from tens of frames per second to hundreds. Also, the line-scan concept utilizing a cylindrical lens was designed to offer a simple yet robust approach to attain high lateral scan speed capacity. Furthermore, in the recent past, electrically tunable lens (ETL),^9^^–^^11^ adaptive lenses,^12^ and digital micro-mirror devices (DMD) have been introduced^13^ as electrical or mechanical means to enhance axial scanning efficiency. The use of a Bessel beam^14^^,^^15^ generated by a conical lens (axicon) was also proposed as an effective approach for achieving volumetric imaging. Although innovative, these methods allow limited improvements in image resolution or acquisition speed that are insufficient for many biomedical studies. Other advanced techniques, such as multiplane imaging by multislits,^16^ reflecting pinholes,^17^ and multiprisms,^18^ have been brought forth to enable simultaneous multidepth acquisition at different axial positions. However, despite the high prospects, such improvements have been limited in the resolution or volumetric imaging rate.

To mitigate such shortcomings, we developed a high-speed, micron-scale resolution volumetric imaging system by implementing a rapid axial scanning unit (via tunable acoustic gradient index of refraction: TAG lens). As the TAG lens can rapidly change its focal length, the penetrating excitation light can be robustly scanned along the axial direction. As in conventional confocal microscopy, the emitted light is also “de-scanned” by the TAG lens, redirecting it to a fixed point on the detector, which allows for high-speed, high-resolution volumetric imaging. In general, the TAG lens enables acceleration of volumetric imaging speed up to tens of hertz,^19^^,^^20^ providing a highly effective means of improving the imaging depth with high-speed axial scan capacity and low optical aberration.

We developed a TAG-based rapid axially scanned and de-scanned (RASAD) line-scan confocal microscopy (LSCM) system by integrating a TAG lens module into LSCM. This study builds upon our previously introduced ETL-based volumetric imaging system,^21^ with the current work focusing on significantly increasing volumetric acquisition speed by replacing the ETL with a TAG lens. This boosts volumetric acquisition rates up to 200 Hz, an improvement of roughly an order of magnitude over the ETL system. Compared to conventional methods, it achieves higher imaging speed than point-scan confocal microscopy and greater penetration depth than wide-field microscopy. We optimized the volume imaging capability using fluorescent microbeads and *ex vivo* samples. To validate the practical utility of the TAG-based RASAD LSCM system, we conducted *in vitro* calcium imaging of neonatal mouse cardiomyocytes and *in vivo* mouse cortical blood vessel imaging. We measured the calcium transients and cell boundary displacement induced by the spontaneous contraction of the cardiomyocyte and quantified the speed of cortical blood flow at multiple depths in the mouse brain, adjusting 2D projection velocities based on 3D geometry for measurement accuracy. Such technical advancements through the optimal implementation of rapid volume imaging would greatly enhance the applicability of 3D fluorescence imaging, including spatiotemporal monitoring of neurophysiological events.

## 2 Materials and Methods

### 2.1 Optical Implementation

**Fig. 1:**
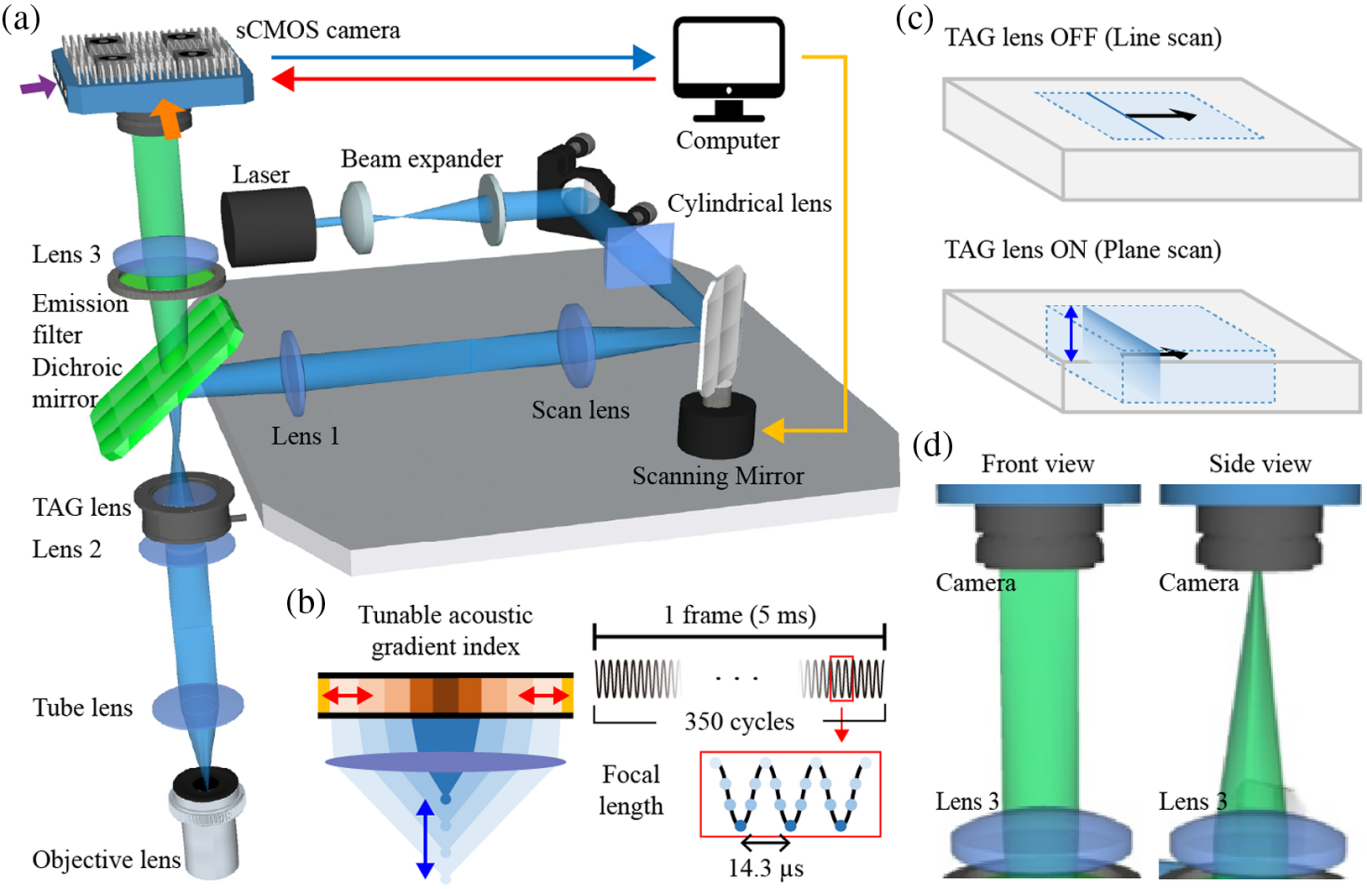
Schematic diagrams of the TAG-based RASAD microscopy system. (a) The experimental setup and beam path. (b) The left panel illustrates the working principle of the TAG lens; the right panel shows the TAG lens oscillation during a single camera frame acquisition along with corresponding changes in focal length. (c) A comparison of 2D line and 3D plane scans with the TAG lens in the off and on states, respectively. (d) Front and side views of the emission beam shape between lens 3 and the camera. The orange and purple arrows in panel (a) indicate the front and side viewing directions, respectively.

For excitation, a 473 nm laser (MBL-III-473, Changchun New Industries Optoelectronics Tech. Co., Ltd.) was used as the light source. The beam was expanded by two lenses (Thorlabs) with focal lengths of 50 and 150 mm, forming a 4-f system. Then it passed through a cylindrical lens (f=50  mm, Thorlabs) before being reflected by a scanning mirror (x-axis of GVS002, Thorlabs). The excitation line produced by the cylindrical lens was rapidly moved vertically by the TAG lens and laterally scanned by the scanning mirror to generate a volume excitation. After being reflected by the scanning mirror, the beam traveled to the scan lens ($f_{\text{scan}}=60\;\mathrm{mm}$) and reached another lens ($f_{L1}=150\;\mathrm{mm}$) for focusing before being reflected by a dichroic mirror (FF552-Di02-25×36, Semrock). Thereafter, the beam propagated to another 4-f system formed by the TAG module (a combination of TAG lens (TAGLENS-T1, Mitutoyo, part no. 02AVB230) and a plano-convex lens ($f_{L2}=250\;\mathrm{mm}$)), and a tube lens ($f_{\mathrm{tb}}=250\;\mathrm{mm}$), thereby optimizing the numerical aperture of the objective lens [Fig. 1(a)]. As the static focal length of the TAG lens is longer than 1000 mm, the plano-convex lens was combined to form a 4-f system. This arrangement was necessary to convert the TAG lens’s weak optical modulation into sufficient axial scanning depth and required a different optical layout compared to our previous ETL-based design.^21^ In principle, the TAG lens is designed to vary the focal length based on the acoustic action of liquid wave interference within the TAG lens, which is generated by the actuation of piezo electrodes that surround the lens [Fig. 1(b)].^22^ The speed of axial focal point transition could be in the kilohertz range, and 70 kHz was used in the current study. At the end of the illumination path, an objective lens (UMPLFLN20XW, Olympus) was placed for image acquisition.

In the detection path, fluorescent signals from the sample traveled back to the 4-f system of the TAG module through the objective lens. When the TAG lens was turned off, a line scan resulted in one 2D image, whereas a 3D projection image was obtained with the TAG lens turned on [Fig. 1(c)]. The signal then went through the dichroic mirror before propagating to the emission filter. At the end of the detection path, we placed an sCMOS camera (PCO.edge 4.2, PCO) with another lens ($f_{L3}=50\;\mathrm{mm}$) placed before the camera to focus the signal photons on the detection plane [Fig. 1(d)]. When the computer routed the signal to the scanning mirror (yellow arrow), synchronized pulse signals were generated simultaneously for the activation of the PCO camera (red arrow). The PCO camera was exposed with a rolling shutter to create a virtual slit for attaining confocal effects as images were recorded in the computer(blue arrow). This virtual slit corresponds to ∼3.9  pixels (1 Airy unit), which is consistent with typical optical configurations used in LSCM.^23^ Upon operating the TAG lens at 70 kHz (i.e., resonant frequency), we achieved volumetric imaging at 200 Hz. For consistency of lateral resolutions at various depths and maintenance of the intensity above 50% of peak intensity, 70% of the maximum TAG lens driving current was selected. More information on the optimization of the TAG lens setting is presented in Fig. S1 in the Supplementary Material.

### 2.2 Determining Point Spread Function Using Fluorescent Beads

To analyze the Point Spread Function (PSF), 0.5  μm fluorescent beads (G500, Thermo Scientific) placed on microscope slides were used. The slide with the fluorescent beads was put on a translation stage (MPC-200, Sutter Instrument) using a sample holder. We measured image resolutions at three different settings: “no TAG lens,” “TAG lens off,” and “TAG lens on.” In the “no TAG lens” setting, the TAG lens was entirely removed from the system. In the “TAG lens off” setting, the TAG lens was attached but not activated. In the “TAG lens on” setting, the TAG lens was activated and operated at 70 kHz. At each setting, we imaged fluorescent beads as a 3D stack with continuous axial layers, where Δz=0.5  μm. We then acquired the cross-sectional profiles of the signal intensity for each fluorescent bead in both the lateral and axial directions. After normalizing the data to a value between 0 and 1, we measured the full width at half maximum (FWHM) for each direction.

### 2.3 *Ex vivo* Imaging of Neurons in a Mouse Brain Slice

We prepared a coronal section of the optically cleared brain from a Thy1-eYFP mouse^24^ for *ex vivo* imaging.^25^ Optical clearing was performed to enable effective imaging of complex structures across depth in the tissue. The sample was mounted on a translation stage and imaged in two settings: “TAG lens off” and “TAG lens on.” In the “TAG lens off” setting, we performed full 3D imaging, capturing 135 axial planes with Δz=1  μm. This fine step size was used to resolve vessel orientations in 3D, which was required for determining tilt angles and calculating actual blood flow velocities. The Temporal-Color Code plugin (ImageJ/Fiji) was utilized to visualize the 3D stack consisting of 2D images.^26^ In the “TAG lens on” setting, we activated the TAG lens at 70 kHz and simultaneously turned on the camera at 150 Hz for 5 s. In this case, each camera frame corresponds to an axial projection formed by a continuous sinusoidal focal sweep across the depth range, rather than discrete z-planes.

### 2.4 *In Vitro* Imaging of Cultured Neonatal Mouse Cardiomyocytes

We isolated cardiomyocytes from neonatal C57BL/6 mice and cultured them in a petri dish for *in vitro* calcium imaging using a procedure adapted from a published protocol.^27^ Fluo-3 AM (F1242, Thermo Fisher Scientific) was used as a fluorescent calcium indicator to monitor calcium transients in cultured cardiomyocytes.^28^ The sample was mounted on a translation stage and imaged in “TAG lens on” setting. The TAG lens was operated at 70 kHz, whereas the camera was simultaneously activated at 30 Hz for 10 s. A higher magnification objective lens (LUMPLFLN40XW, Olympus) was placed for image acquisition to see the contractions more clearly. Calcium transients of cardiomyocytes were dynamically captured and processed to calculate ΔF/F. From the fluorescence intensities extracted for each region of interest, the baseline fluorescence ($F_{0}$) was determined as the average fluorescence during a quiescent period. Changes in fluorescence were visualized by calculating $\Delta F/F=(F_{t}-F_{0})/F_{0}$, where $F_{t}$ represents the fluorescence intensity over time. Cell boundary displacement induced by the spontaneous contraction was quantified by extracting edge positions from 5 kymographs per region, generated along lines perpendicular to the local cell boundaries. Extracted edge positions were averaged to produce representative displacement profile.

### 2.5 *In Vivo* Imaging for Assessing Cerebrovascular Dynamics in the Mouse Brain

We prepared a 5 mm diameter cranial window in the skull of a C57BL/6 mouse to evaluate cerebrovascular activities using a procedure adapted from a published protocol.^29^ We administered Zoletil/Xylazine (60/10  mg/kg body weight) mix diluted with saline solution intraperitoneally for anesthesia during surgical procedures and imaging. The whole process was done under the body temperature control at 37.0°C to 37.5°C using a heating plate system (CU-201, Live Cell Instrument, South Korea). We performed the experiment in two settings: “TAG lens off” and “TAG lens on.” In the “TAG lens off” setting, we acquired a full volume consisting of 100 images with Δz=1  μm. In the “TAG lens on” setting, we obtained full volumetric images of the same field-of-view (FOV) with the TAG lens running at 70 kHz and the camera running at 200 Hz (or 150 Hz) for 5 s.

To evaluate the blood flow velocity from 3D vessel geometry, we translated the method of measuring blood flow velocity from 2D projection images to 3D. We measured the kymograph angle θ to estimate the flow velocity of each blood vessel. This involved applying the Sobel filter to remove artifacts and performing a Radon transform (i.e., kymograph to velocity).^30^ We then quantified the true flow velocity along the vessel, considering the 3D architecture of vessel geometry, in which the corrected velocity ($v_{c}$) was calculated from the projected velocity ($v_{p}$) acquired from 3D images, using the following equation:

$$v_{c}=v_{p}/\cos\;\Phi .$$ (1)

Here, Φ (deg) denotes the measured angle of the blood vessel with respect to the objective lens surface (Fig. S3 in the Supplementary Material). By calculating the FWHM value of the cross-sectional profile^31^ and averaging diameters from five evenly spaced cross-lines as shown in Fig. 5(f), we measured the diameter of the blood vessels.

All animal experiments were conducted in accordance with the guidelines of the Institutional Animal Care and Use Committee (IACUC) of the Gwangju Institute of Science and Technology (GIST), under approved protocol number [GIST-2021-111]. All procedures adhered to the ARRIVE guidelines for the care and use of laboratory animals. Mice were anesthetized throughout all surgical and imaging procedures using an intraperitoneal injection of a Zoletil/Xylazine mixture (60/10  mg/kg body weight) diluted in saline.

## 3 Results

### 3.1 Image Resolution of RASAD Microscopy

**Fig. 2:**
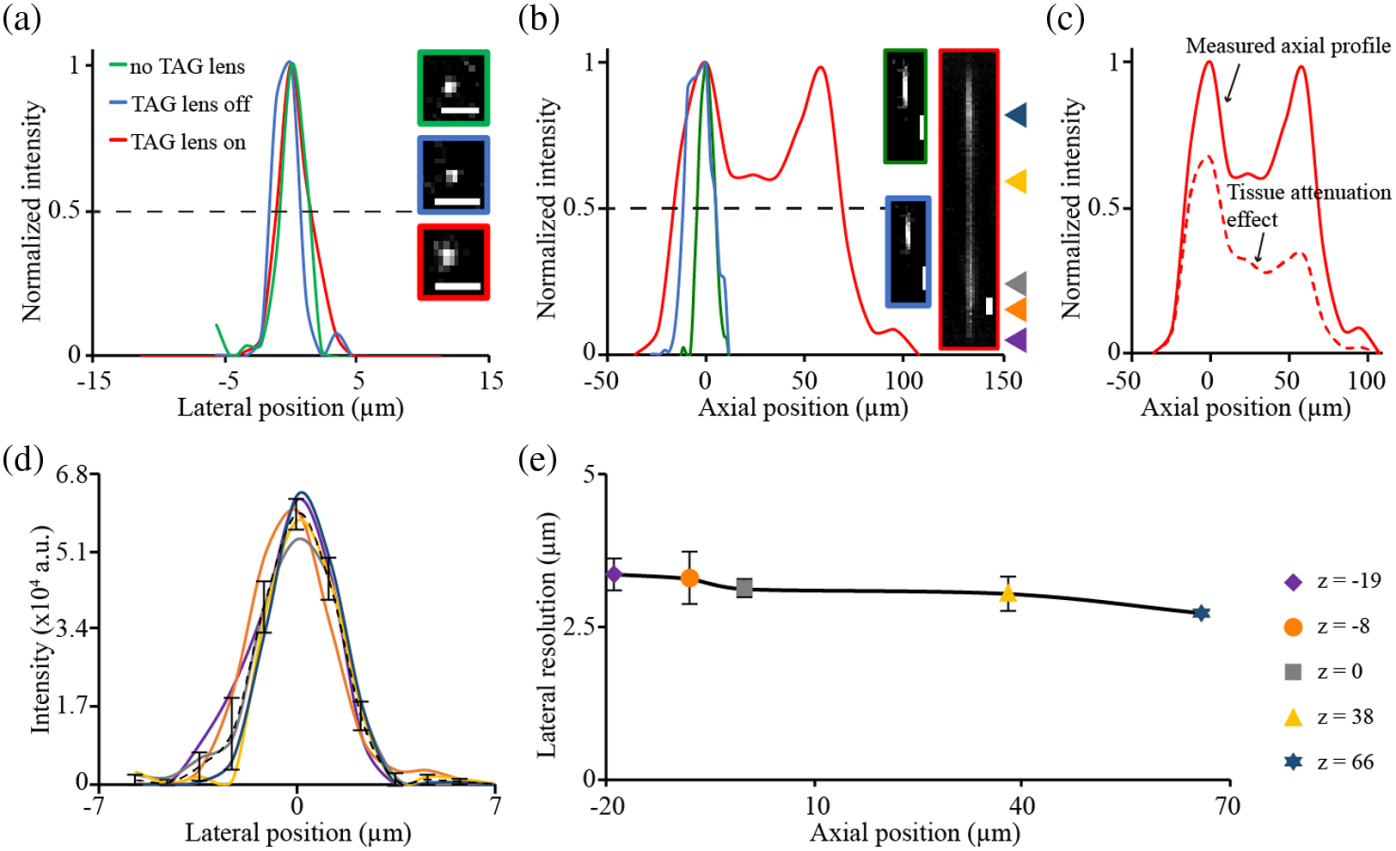
Evaluation of RASAD point spread function (PSF) on lateral (XY) and axial (XZ) planes. (a) Lateral PSF profiles for three TAG lens settings: “no TAG lens,” “TAG lens off,” and “TAG lens on.” (b) Axial PSF profiles for the same three settings. (c) Estimated axial profile showing the tissue attenuation effect (dashed line, $\mu =0.00976\;\mu m^{-1}$), based on the measured profile in panel (b) for the “TAG lens on” setting. (d) Lateral PSF profiles obtained at different axial depths with the TAG lens activated at 70 kHz. The dashed line indicates the averaged profile. (e) Lateral resolutions measured from PSFs at different depths along the axial direction. Scale bar: 5  μm.

We calculated the FOV of detected images using the USAF target, which was $450\times 450\;\mu m^{2}$, and acquired the PSF in three imaging settings: (1) no TAG lens, (2) TAG lens off, and (3) TAG lens on. From these values, we evaluated the lateral and axial PSF. By measuring the FWHM value of the cross-sectional signal intensity profile, we found that the measured lateral resolution was 1.9±0.2  μm for the “no TAG lens” condition and 2.1±0.1  μm for the “TAG lens off” condition [Fig. 2(a)]. Furthermore, the axial FWHM values were 10.8±0.5  μm and 15.2±1.3  μm for the “no TAG lens” and “TAG lens off” conditions, respectively. Note that the aberration caused by adding a TAG lens module in the system was modest. At the “TAG lens on” setting, the measured lateral resolution was 2.6±0.2  μm, whereas the depth of focus greatly increased to 135.7±2.8  μm [Fig. 2(b)], confirming the successful elongation of the axial PSF for enabling rapid volumetric imaging.

To estimate the axial profile under *in vivo* conditions, we applied an exponential decay model of the form $e^{-\mu z}$ to the measured axial profile, where an attenuation coefficient of $\mu =0.00976\;\mu m^{-1}$ and z is the depth from the tissue surface. Based on experimental measurements in the mouse cerebral cortex at 453 nm,^32^ this value provides a conservative estimate for visible-light attenuation in brain tissue. As shown in Fig. 2(c), the resulting profile exhibits reduced intensity at deeper focal positions while preserving the overall PSF structure, indicating stable axial performance of the TAG lens under *in vivo*-relevant conditions.

To evaluate the stability of lateral image resolution, we examined FWHM values of the lateral PSFs collected from five different depths during the activation of the TAG lens (i.e., “TAG lens on”). Through analyses of the ratios between peak intensity levels across different axial layers [Fig. 2(d)], we observed that these ratios fell within the range of 85% and 100%. This finding confirms the robust functioning stability of the TAG lens module. Furthermore, the average FWHM value was 2.5±0.1  μm, suggesting that the elongation of the axial PSF did not generate appreciable changes in the lateral image resolution across different axial positions [Fig. 2(e)].

### 3.2 Volumetric Imaging of *Ex Vivo* Cleared Mouse Brain

**Fig. 3:**
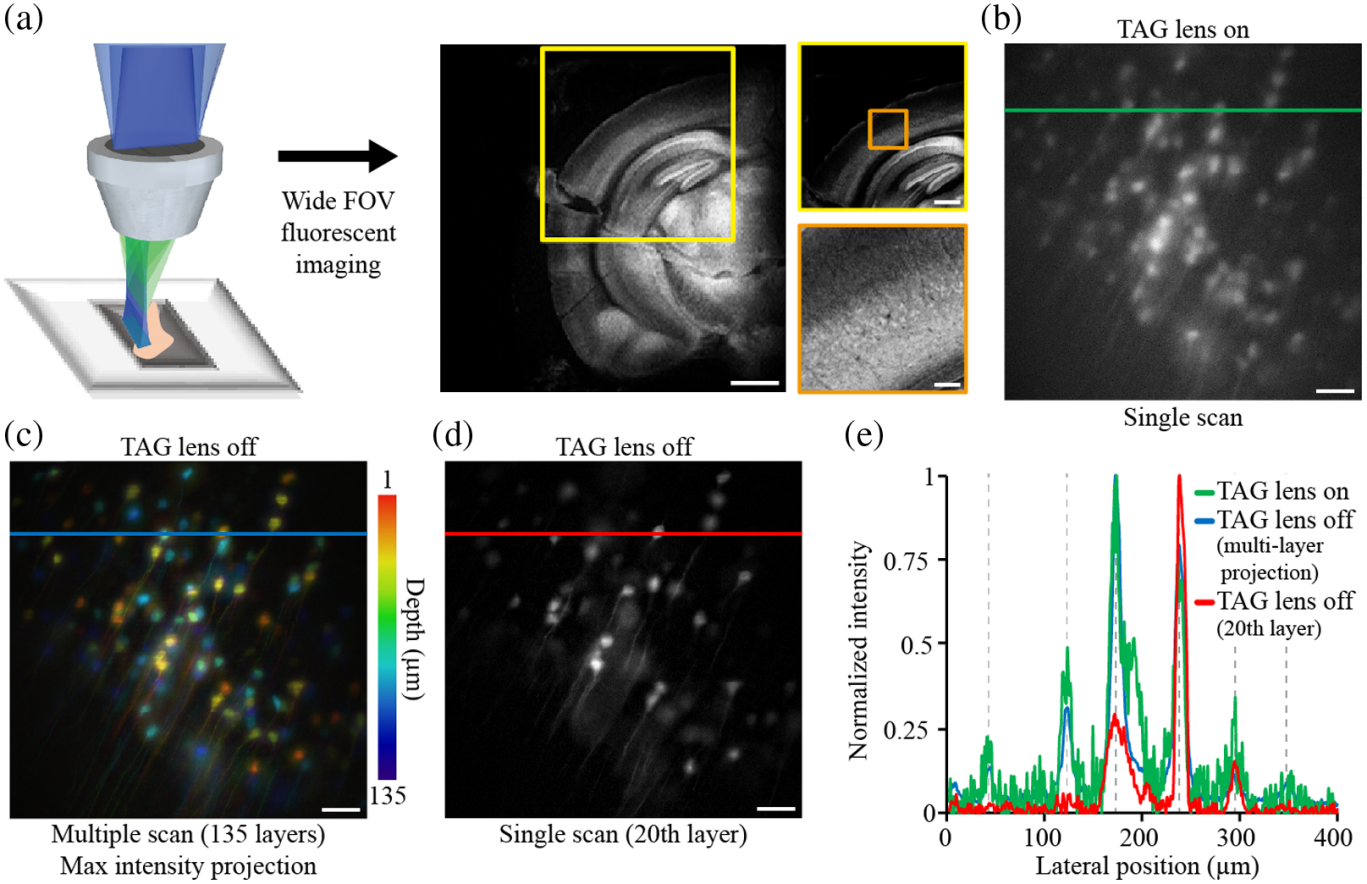
*Ex vivo* brain slice neuron imaging using TAG-based RASAD. (a) Schematic of the system and images of an *ex vivo* cleared brain slice: neurons in the cleared brain slice under a wide FOV fluorescence microscope. Scale bars: 1 mm, 500  μm, and 100  μm, respectively. (b) Volumetric image generated with the TAG lens on. (c)–(d) Stack images generated with the TAG lens off for volumetric imaging with a depth scan step size of 1  μm, where (c) maximum intensity projection of the full stack and (d) image from a single layer are shown. (e) Normalized cross-sectional profiles from the same FOV as (b)–(d). Scale bar: 50  μm.

An experimental schematic for imaging the cleared *ex vivo* brain and images acquired with a wide FOV fluorescence microscope are shown in Fig. 3(a). To identify the positions of neurons, we first captured the image of brain slices using wide FOV fluorescence microscopy. Performance comparison of TAG-based RASAD and wide-field microscopy is presented in Fig. S2 in the Supplementary Material. We then activated the TAG lens for “TAG lens on” volumetric acquisition [Fig. 3(b)], after which LSCM was performed for “TAG lens off” by axially scanning 135 layers with a step size of 1  μm. Both “on” and “off” settings used the same volumetric FOV. Cross-sectional profiles were obtained from these images: (1) a single image obtained with the “TAG lens on” [Fig. 3(b)], (2) maximum intensity projection of the image stack obtained with the “TAG lens off” [Fig. 3(c)], and (3) a single layer scanned with the “TAG lens off” [Fig. 3(d)]. The cross-sectional profile of the single scan with the “TAG lens on” [green line in Fig. 3(e)] was nearly identical to that obtained from the multiple-layer scan with the “TAG lens off” [blue line in Fig. 3(e)]. As expected, the signal profile from a single-layer scan with the “TAG lens off” [red line in Fig. 3(e)] resulted in a 2D subset of the volume image signal, with some neuronal signals absent compared to the one with the “TAG lens on” [green line in Fig. 3(e)]. Consequently, the addition of the TAG lens (thus TAG lens-based RASAD microscopy) enabled rapid volumetric imaging, greatly enhancing the acquisition efficiency of the typical LSCM system while minimally sacrificing the image quality.

### 3.3 Volumetric Imaging for Monitoring Spontaneous Contractions in Cardiomyocytes

**Fig. 4:**
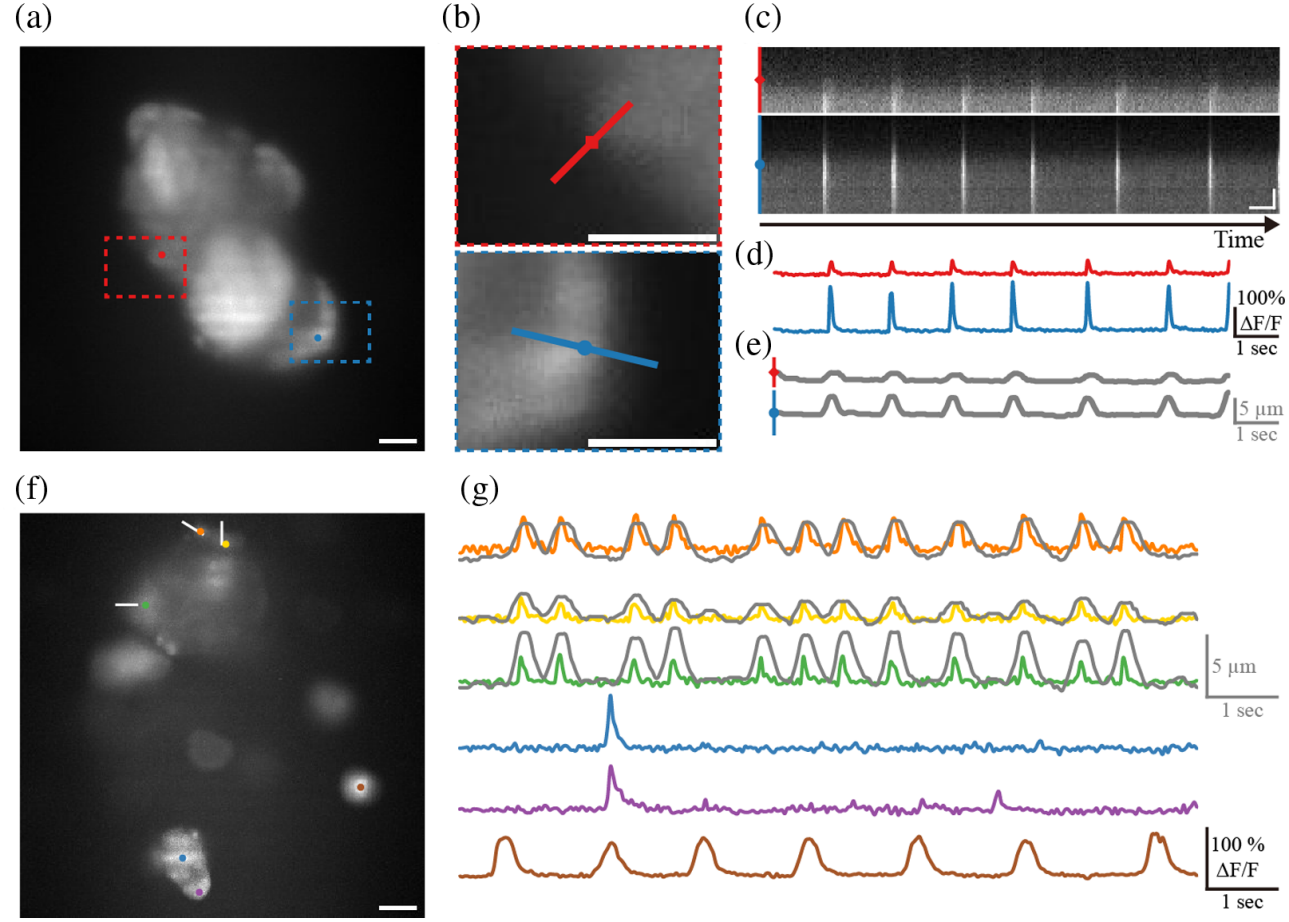
*In vitro* cardiomyocytes imaging using the TAG-based RASAD system. (a) Full FOV of the cultured neonatal mouse cardiomyocyte imaging for calcium changes and contraction analysis. (b) Magnified images showing the region denoted by the dashed boxes in panel (a), with the kymograph line overlaid for measuring cell boundary displacement. (c) Kymograph profiles obtained from the line of each image in panel (b). (d) Transient calcium changes (ΔF/F) of 2 points marked with color dots in FOV. (e) The contraction-induced cell boundary changes extracted from the kymograms in panel (c). (f) Another full FOV of the cultured neonatal mouse cardiomyocytes imaging. (g) Transient calcium changes (ΔF/F) of 6 points marked with color dots in panel (f). The contraction-induced cell boundary changes calculated from white kymograph lines in panel (f) are drawn in gray, overlapping the calcium graph. Scale bars: 20  μm in panels (a), (b), and (f); 1 s and 10  μm in panel (c) (Video 1, MP4, 672 KB [URL: https://doi.org/10.1117/1.NPh.12.4.045013.s1]; Video 2, MP4, 4.69 MB [URL: https://doi.org/10.1117/1.NPh.12.4.045013.s2]).

In an *in vitro* setting, functional imaging was performed using the TAG-based RASAD system at a camera frame rate of 30 Hz over a 10-s period. Spontaneous contractions of neonatal cardiomyocytes and associated calcium fluctuations were captured (see Videos 1 and 2). From a large cell aggregate, two regions were selected for fluorescence analysis [Fig. 4(a)]. Cell boundary changes near each region were extracted from kymograms generated along lines oriented perpendicular to the direction of displacement [Figs. 4(b)–4(c)]. For each region, five closely spaced lines were used. Figures 4(d)–4(e) present boundary displacement and corresponding calcium transients plotted on the same time scale. The synchronized changes confirm that the entire aggregate contracts simultaneously, as reflected in both calcium dynamics and boundary shifts.

In another FOV containing multiple cell aggregates, six points were chosen to measure calcium transient, and boundary displacements were analyzed near three of these points on a larger aggregate [Fig. 4(f)]. Figure 4(g) shows the extracted calcium transients and boundary displacements along the white lines in Fig. 4(f). The results indicate that although cells within the same aggregate contract in synchrony, different aggregates exhibit independent contraction patterns.

### 3.4 Fast Volumetric Imaging of Blood Vessels in the Mouse Brain

**Fig. 5:**
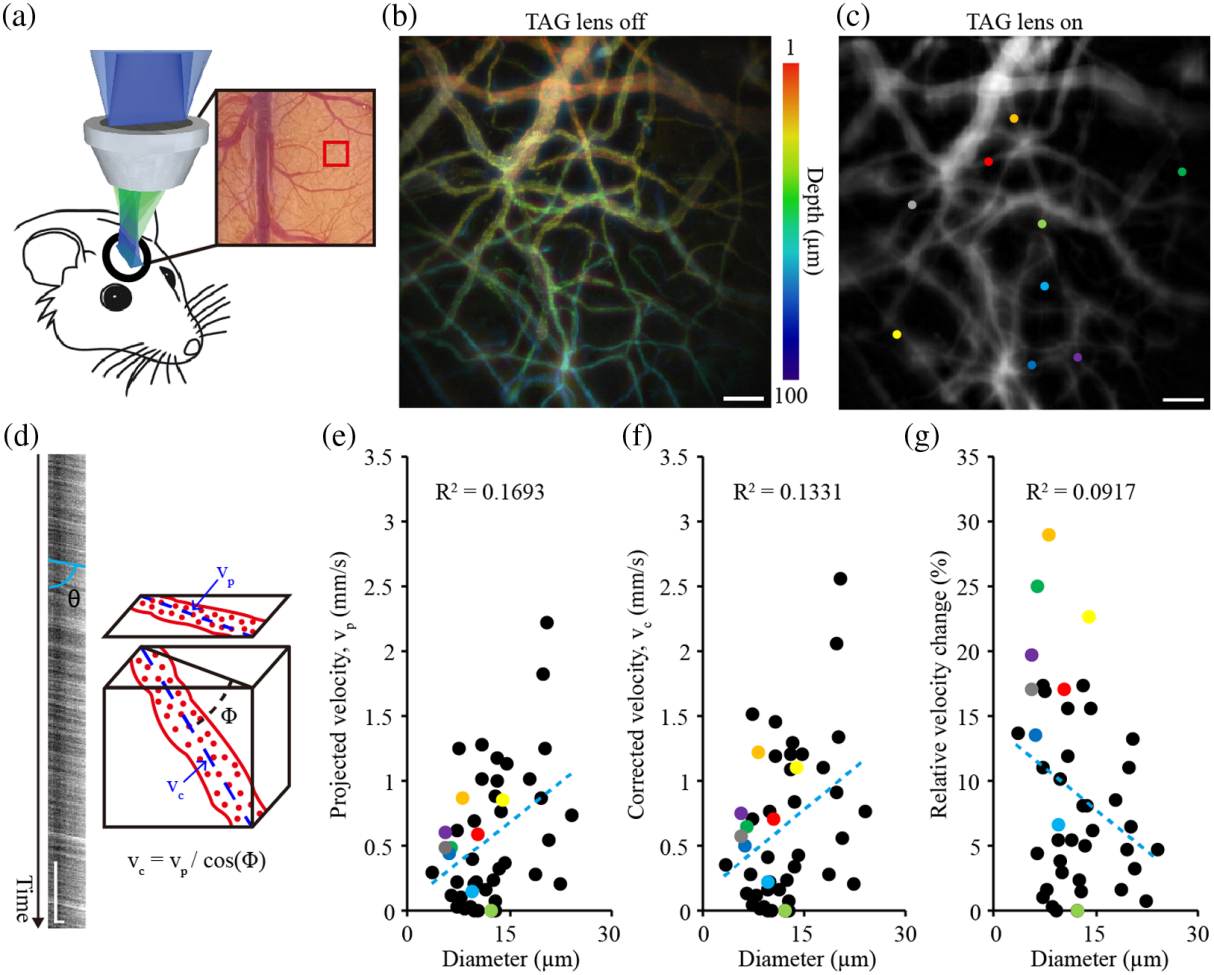
Quantitative *in vivo* blood vessel imaging using the TAG-based RASAD system. (a) Schematic of the system for imaging the mouse brain through a cranial window. (b) Full-stack volumetric image acquired with the “TAG lens off” and a depth scan step size of 1  μm. (c) Volumetric images acquired with the “TAG lens on.” (d) The mean blood flow velocity based on the kymograph angle θ and projection angle Φ. (e) Relationship between the projected blood flow velocity ($v_{p}$) and vessel diameter for 47 data samples. (f) Relationship between the corrected velocity ($v_{c}$) and vessel diameter. (g) Relative velocity differences, i.e., $(v_{c}\text{-}v_{p})/v_{c}\times 100\%$, plotted against vessel diameter. Scale bars: 50  μm in panels (b) and (c); 0.5 s and 10  μm in panel (d).

A schematic for monitoring the *in vivo* blood vessels is depicted in Fig. 5(a), which was used to perform quantitative cerebrovascular imaging. Full volume imaging with the “TAG lens off” was conducted for the depth range of 0 to 100  μm with a step size of 1  μm, as shown in Fig. 5(b). Once the TAG lens was activated at 70 kHz, the same 3D FOV was successfully scanned at the camera frame rate of 150 Hz for 5 s [Fig. 5(c)].

Figure 5(e) illustrates the uncorrected (i.e., projected: $v_{p}$) blood flow velocity values plotted against the diameters of the sampled blood vessels (n=47). The blood flow velocity in 3D space was estimated by adjusting the velocity value calculated based on kymograph angle θ using Eq. (1) to obtain the corrected velocity ($v_{c}$) [Fig. 5(d)]. As a result, the highest flow velocity was 2.58  mm/s, whereas the largest diameter was 24.1  μm [Fig. 5(f)]. A comparison between $v_{p}$ and $v_{c}$ showed that the corrected velocity values significantly differed from those calculated using the conventional projection method. Interestingly, the correlation between velocities and the vessel diameter (i.e., slope) decreases when using the corrected velocity [Figs. 5(e), 5(f)]. Our finding of no significant correlation between blood flow velocity and vessel diameter within the examined range is consistent with a previous study that also observed a weak correlation within the small diameter range.^33^ Upon assessing the degree of velocity correction, we found that the relative change, calculated as $(v_{c}\text{-}v_{p})/v_{c}\times 100$ (%), could reach up to 30% [Fig. 5(g)].

### 3.5 Fast Volumetric Imaging for Classification of Blood Vessel Types

**Fig. 6:**
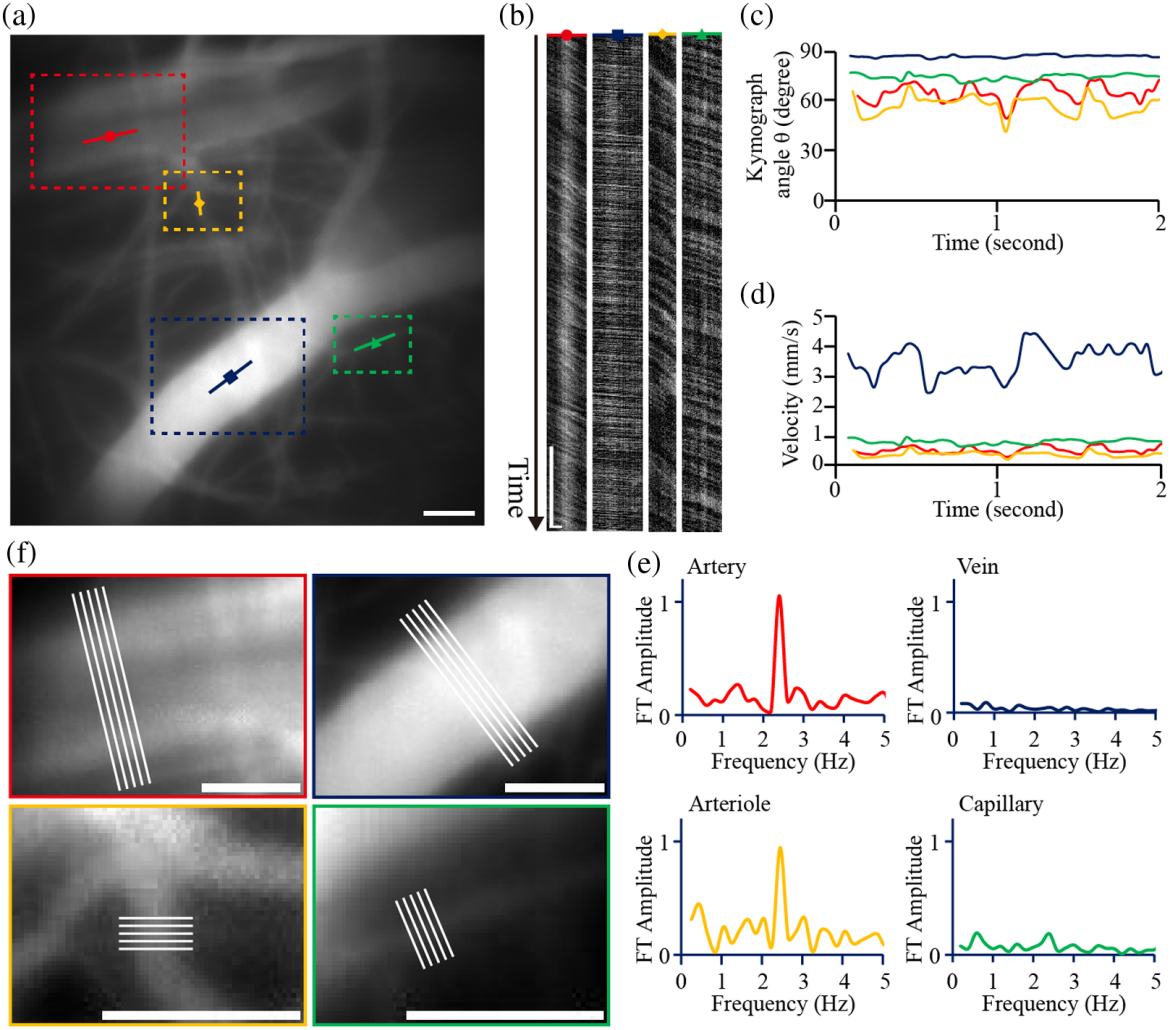
*In vivo* blood vessel imaging and analyses using the TAG-based RASAD system. (a) Full FOV of the blood vessel imaging recorded. (b) Kymograph profiles of the vessels for red, blue, yellow, and green arrows in FOV. (c) The analyzed angle θ of the chosen blood vessels for 2 s. (d) Calculated blood flow velocity of each vessel for 2 s. (e) Frequency distribution of four chosen vessels via Fourier transform analysis. (f) Magnified images of the vessels with five cross-sectional lines used for measuring the diameters of the vessels denoted by the red, blue, yellow, and green dashed boxes in panel (a). Scale bars: 50  μm in panels (a) and (f); 0.5 s and 20  μm in (b) (Video 3, MP4, 109 KB [URL: https://doi.org/10.1117/1.NPh.12.4.045013.s3]).

Using the *in vivo* setting, full volumetric imaging was conducted using the TAG-based RASAD system at the camera frame rate of 200 Hz (see Video 3). For comparison, four blood vessels with different kymograph angles were chosen for analysis [Figs. 6(a)–6(d)]. Among these, the kymograph angles of the two vessels marked in red and yellow were 65.4±4.9 and 58.0±6.7  deg, respectively. These vessels exhibited periodic fluctuations that were likely caused by the heartbeat. For the vessels marked with blue and green, kymograph angles were 86.6±0.8 and 73.7±1.6 (deg), respectively, and they did not exhibit periodic fluctuations [Fig. 6(c)]. Furthermore, the Fourier transform of the time-dependent kymograph angle θ(t) was performed to illustrate the frequency traits of blood flow in each vessel [Fig. 6(e)]. As a result, no appreciable peaks were detected for the $\mathrm{FT}[\theta_{\text{blue}}(t)]$ and $\mathrm{FT}[\theta_{\text{green}}(t)]$, whereas robust frequency peaks at ∼2.4  Hz were observed for $\mathrm{FT}[\theta_{\text{red}}(t)]$ and $\mathrm{FT}[\theta_{\text{yellow}}(t)]$, which is much lower than the typical mouse heart rate at ∼10  Hz. It should be noted that the animal was anesthetized with a Zoletil/Xylazine mixture. The observed reduction in heart rate is most likely attributable to Xylazine, an α2-adrenergic agonist known to induce a slower heart rate through inhibition of the sympathetic nervous system.^34^^,^^35^

Upon quantifying the flow velocities ($v_{c}$) as shown in Fig. 6(d), the calculated values [Eq. (1)] for the vessels that did not show apparent frequency peaks were 3.64±0.14  mm/s for the vessel marked in blue and 0.95±0.08  mm/s for the one marked in green. For those exhibiting beats (i.e., yellow and red), flow velocities were 380.6±79.6  μm/s and 521.5±111.6  μm/s, respectively. The diameter of each blood vessel was averaged from the FWHM values obtained from the five cross-sectional lines, as shown in Fig. 6(f), in which the vessel diameters marked in blue, green, yellow, and red were 66.4, 6.9, 12.2, and 82.8  μm, respectively.

The type of each vessel was estimated by considering its diameter, flow velocity, and flow fluctuation caused by heart pulsation. We identified the vessel marked with red in Fig. 6 as an artery due to its large diameter and the presence of putative pulsatile fluctuations. Similarly, the vessel marked with yellow, which had a much smaller vessel diameter and noticeable periodic fluctuations, was identified as an arteriole. The vessel marked in blue, with its large diameter and significant kymograph angle, was classified as a vein. Finally, the vessel marked in green, which had the smallest diameter and a relatively smaller kymograph angle than the others, was classified as a capillary.

## 4 Discussion and Conclusion

By incorporating LSCM with a multifocal optical module based on the TAG lens, we have successfully implemented a high-speed volumetric optical imaging system. In schematics, LSCM ensured the lateral resolution stability while adding a TAG lens significantly extended the PSF profile in the axial (depth) direction. Integrating these strategies extended the hypothetical maximum focal depth to 135  μm. Implementing a TAG lens within a 4-f relay introduces a degradation in lateral resolution, reflecting a trade-off for achieving rapid axial scanning and extended depth of field.^36^^,^^37^ Nevertheless, with careful alignment and synchronized detection, TAG-enabled sectioning can enhance image contrast and the detectability of fine structures in thick specimens. In practice, the imaging depth of ∼100  μm was achieved with the maximum acquisition rate of 200 Hz while preserving the lateral resolution of ∼2.6  μm across the whole FOV. Although successfully designed and implemented, we were yet unable to demonstrate the axial resolution using the TAG-based RASAD system as the signals from all depths converged in a single frame (Fig. S4 in the Supplementary Material), a trade-off made to enhance the speed of volumetric imaging.

Previously, the extension of the depth of focus was proposed through various system modifications such as reducing the numerical aperture of the objective lens for illumination,^16^ adding either an ETL^21^^,^^38^ or a piezo motor unit,^39^ and using either an adaptive lens^12^ or a deformable mirror.^40^^,^^41^ However, most approaches designed to improve axial scan capacity become limited in the image resolution or resultant imaging speed. On the other hand, the remote focusing method was used to maintain the lateral resolution in rapid volumetric imaging.^42^^,^^43^ However, the achievable acquisition speed is still in need of much improvement to effectively capture key dynamic features in biological and physiological activities. Another confocal volumetric imaging method, the multi-z strategy,^17^ was devised to enhance the sampling density and image acquisition rate. Although highly effective, this method faces limitations with the acquisition rate. In response to the shortcomings of such previous attempts, this study aims to offer a detailed volumetric representation of neural structures and a high frame rate imaging method for quantifying cerebrovascular events.

As a result of implementing the TAG-based RASAD system, we achieved a depth of focus of 135  μm at 70% of the maximum driving current of the TAG lens. Increasing the driving current up to 100% could, in principle, extend the axial focal displacement toward ∼200  μm; however, our measurements (Fig. S1 in the Supplementary Material) indicate that this elongation is accompanied by intensity falloff in the central region. Despite the improved axial scanning range, the observed uneven distribution of the axial PSF profile is likely due to the sinusoidal characteristic of the driving current. In addition, signal attenuation becomes more pronounced under *in vivo* conditions due to tissue scattering and absorption. Technically, although further studies are warranted, challenges related to depth-dependent signal reduction and uneven axial PSF profile can potentially be resolved by dynamically modulating the laser intensity during axial scanning. This can be implemented using an electro-optic modulator (EOM), such as a Pockels cell,^44^ synchronized with the TAG lens to compensate for both the sinusoidal scan profile and depth-related signal decay. Alternatively, subdividing and shaping the laser pulses over time^45^^,^^46^ or modulating the laser power using an acousto-optic modulator may enhance axial uniformity. However, due to the interaction between the laser and tissue, which causes a reduction in the SNR, our TAG-based RASAD was limited to the maximum effective imaging depth of ∼100  μm. Hypothetically, a multiphoton technique with longer wavelength excitation should enable imaging of deeper tissues and structures.^47^ Particularly, integration of the multiphoton imaging and augmenting the driving current would potentially provide a greater imaging depth with the axial focal displacement extending over 200  μm.

In our current study, *in vitro* and *in vivo* experiments were used to demonstrate the practical utility of the TAG-based RASAD system. We successfully captured ΔF/F dynamics of calcium signaling and quantified the cell boundary displacement, thus validating its capability for functional imaging of cellular processes. Although our previous ETL-based system demonstrated basic volumetric imaging, its limited scan speed (∼20  Hz) precluded the capture of rapid biological dynamics.^21^ By contrast, integration of a TAG lens increased the volumetric imaging speed by over tenfold, enabling functional analyses that were not attainable with the ETL configuration. Specifically, for the imaging speed, we performed volumetric imaging at a rate of up to 200 Hz, which is usually sufficient for assessing the cerebral blood flow velocity in pial vessels and parenchymal capillaries. The analysis revealed a weak correlation between vessel diameter and flow velocity within the small diameter range of micro-vessels; nevertheless, vessel types (veins, capillaries, and arteries) were estimated based on their structural and physiological traits. In addition, heartbeat-induced flow fluctuations in arterioles (∼2.4  Hz) were detected, highlighting the system’s potential for studying cerebrovascular dynamics and the influence of biomedical interventions on vascular flows.

In addition, several improvements are conceivable to further enhance the performance of the TAG-based RASAD system. The magnification (scanning range) of the TAG lens can be further optimized by relocating the TAG lens to a different position (e.g., the back focal plane of the objective lens or between two relay lenses), as previously discussed in the report of ETL implementation.^48^ Although both ETL and TAG lens shift the focal length, a faster focal length transition of the TAG lens enabled a much higher image acquisition rate.^21^ Notably, the TAG-based RASAD system is not restricted by the numerical aperture, allowing for easy adaptation of objective lenses for various purposes, such as extending the depth-of-field^49^ or enlarging the lateral FOV.^50^ Moreover, adjusting the active pixel region of the sCMOS to a line or adopting linear photodetector arrays can improve the volumetric image acquisition rate.^51^

Despite the numerous advantages, the current TAG-based RASAD system may suffer from marginal but appreciable image aberrations, and hence an implementation of adaptive optics^52^ is recommended as a rectification option. Also, to provide higher image resolutions, the RASAD system can be further modified to enhance imaging contrast via polarization^53^ or a temporal focusing technique.^54^ In addition, FACED^55^^,^^56^ using a pulsed laser with mechanical manipulations of the excitation source could be applied to boost the lateral scanning speed of RASAD up to a thousand frames/second, whereas the lateral image resolution can be enhanced with confocal microscopy. On the other hand, for physiological measures, the method for assessing the blood flow velocity by kymograph angle θ, as used in the current study, could be improved by adopting spatiotemporal correlation of the optical signals (i.e., speckles) from the RBC movements within blood vessels to determine the velocity fields.^57^ This quantification strategy is likely advantageous for providing a detailed structural depiction of associated blood vessels for each measurement.^58^ Nonetheless, the RASAD system, capable of capturing projection volume images at ∼200  Hz, should be fully applicable to studies involving calcium channel activities in neurons^15^ and voltage-sensitive imaging of neuronal action potentials,^59^ which would require a minimum image acquisition rate of 100 Hz.

In conclusion, we have enhanced the volumetric imaging capability of fluorescence microscopy through the implementation of the TAG-based RASAD system, successfully delivering high-speed, high-resolution images with extended depth of focus that surpass other existing methods. This promising tool has significant potential for wider application in preclinical studies, leading to a better understanding and assessment of underlying mechanisms, diagnosis, prognosis, and treatment strategies of various biomedical disorders.

## Supplementary Material

10.1117/1.NPh.12.4.045013.s01

10.1117/1.NPh.12.4.045013.s1

10.1117/1.NPh.12.4.045013.s2

10.1117/1.NPh.12.4.045013.s3

## Data Availability

Data underlying the results presented in this paper are not publicly available at this time but may be obtained from the authors upon reasonable request.
