# Supplementary material for: Rapid axially scanned and de-scanned line-scan confocal microscopy with a tunable acoustic gradient index of refraction lens for high-speed volumetric in vivo imaging
Source: Neurophotonics. 2025 Dec 22;12(4):045013. doi: 10.1117/1.NPh.12.4.045013 (PMC12721343; doi:10.1117/1.NPh.12.4.045013)
Supplement: Supplementary file 1 [file NPh_012_045013_SD001.pdf]

Rapid axially scanned and de-scanned (RASAD) line-scan confocal microscopy with a TAG lens for high-speed volumetric *in vivo* imaging: Supplemental Material

1 Optimization of TAG Lens for Enhanced Lateral and Axial Resolution in TAG-based RASAD

The TAG lens can be activated by a driving current ranging from 70 % to 100 % of amplitude, resulting in a shift in focal length. The PSF's FWHM value was measured at 70 %, 80 %, 90 %, and 100 % to determine the proper current range for the experiment (Fig. S1). At 70 % amplitude, the lateral resolution (FWHM value) was 2.6 ± 0.2 µm, while at 80 % of amplitude, it was 2.4 ± 0.1 µm. The lateral FWHM values at 90 % and 100 % amplitude were 2.4 ± 0.1 µm and 2.9 ± 0.1 µm, respectively. Regarding axial resolution, the system achieved greater depth as the TAG lens amplitude increased. At each amplitude, the axial resolutions were: 135.7 ± 2.8 µm (70 %), 146.7 ± 1.5 µm (80 %), 171.9 ± 1.5 µm (90 %), and 186 ± 3.1 µm (100 %).

However, the experimental results showed non-uniform intensity distribution in the axial direction as the amplitude increased. At higher amplitudes (90 % and 100 %), there was a significant decrease in intensity (below 0.5) in the middle of the axial direction, causing the loss of imaging signal in that specific volume. Furthermore, by investigating the lateral FWHM value across different layers, the TAG lens at 70 % amplitude provided the most optimized value of 2.5 ± 0.1 µm. Meanwhile, the lateral FWHM values at 80 %, 90 %, and 100 % amplitudes were 3.1 ± 0.3 µm, 2.7 ± 0.3 µm, and 3.0 ± 0.1 µm, respectively.

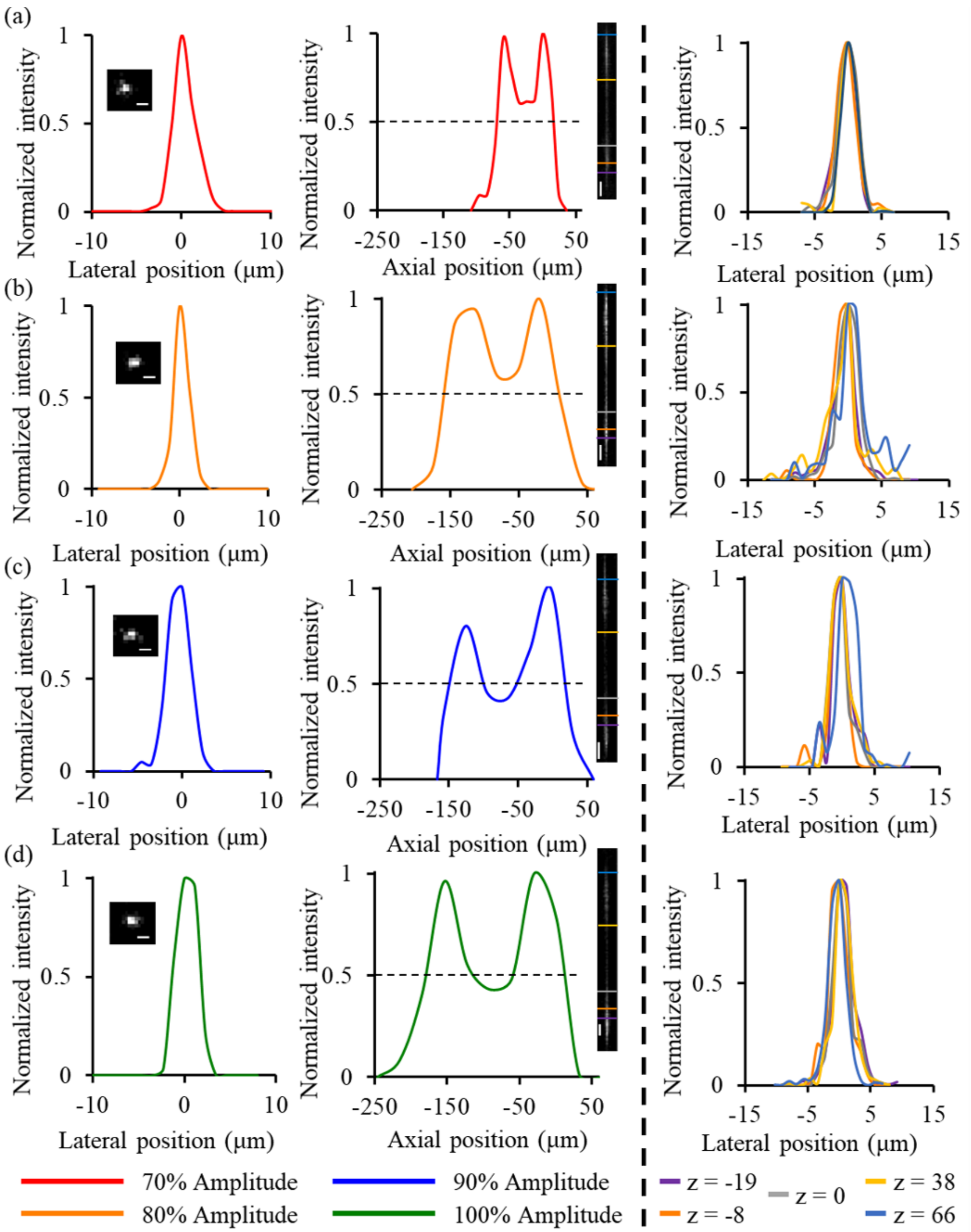

Fig. S1 The PSF measurement at four driving currents (70 %, 80 %, 90 %, and 100 %) of TAG lens in lateral (XY) and axial (XZ) direction: PSF of lateral direction (left), PSF of axial direction (middle), and PSF at five different depths along the axial direction (right) when the TAG lens was activated at (a) 70 %, (b) 80 %, (c) 90 %, and (d) 100 % amplitude. Scale bar: 5 µm.

2. Comparison of Image Quality: TAG-based RASAD vs. Wide-Field Imaging

To highlight the superiority of TAG-based RASAD, image quality was compared between two models: TAG-based RASAD and wide-field imaging with *ex vivo* cleared brain slice sample. Images from both models were captured at the same location and under identical conditions. The TAG lens was activated at 70 % amplitude with a camera speed of 150 fps (Fig. S2a, c). We analyzed the normalized intensity data of the cross-sectional profile at the same position in both images (Fig. S2b, d). The comparison revealed that the peak intensities of the two cross-sectional profiles of images taken from wide-field imaging were at least half lower than that in the TAG-based RASAD. This result indicates that TAG-based RASAD outperforms conventional imaging systems in contrast and information.

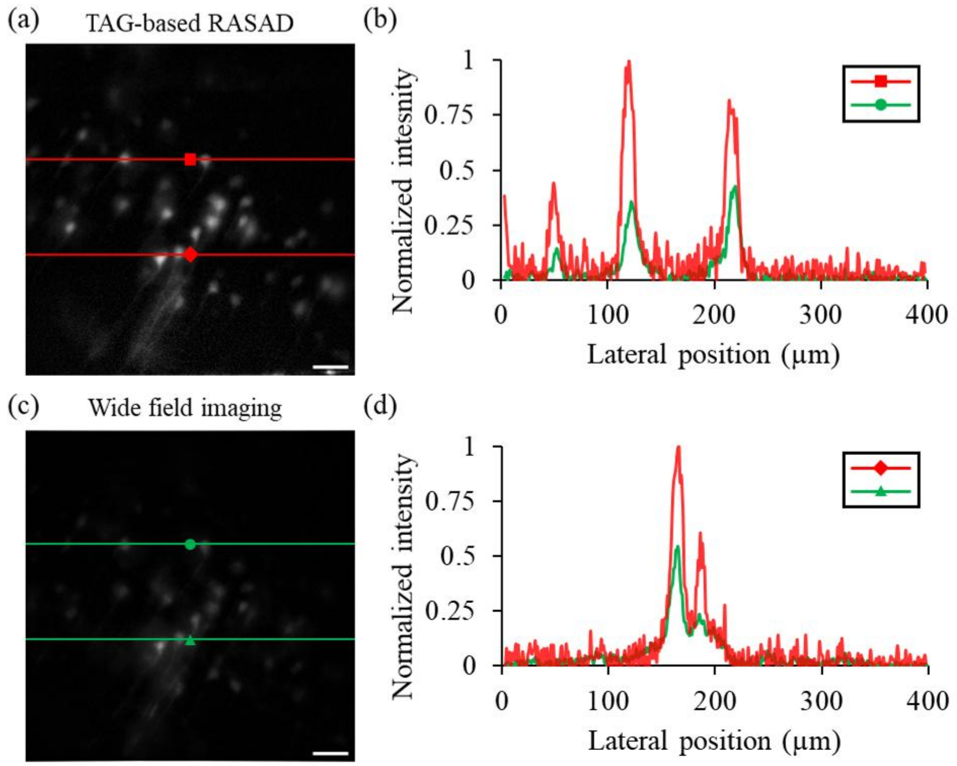

Fig. S2 The performance comparison of TAG-based RASAD and wide field fluorescent microscopy: (a) and (c) The image of the *ex vivo* sample at the same FOV acquired from each imaging system. (b) and (d) The cross-sectional intensity profiles at the same positions. Scale bar: 50 μm.

3. Quantitative Measurement of Blood Vessel Properties and Tilt Angle Calculation

The research involved quantitative measurement of blood vessel properties such as vessel diameter and blood velocity. The FWHM values obtained from the cross-sectional profiles perpendicular to the vector of the blood vessel (red lines in Fig. S3b) were used to calculate vessel diameter. For calculating the mean blood vessel velocity, we calculated the kymograph angle θ based on the kymograph profile using a line parallel to the blood vessel direction vector (Fig. S3b, c). However, since blood vessels exist in three dimensions, the actual velocity may vary according to the tilt angle Φ of the blood vessel relative to the projected image from TAG-based RASAD. The actual tilt angle Φ was calculated based on the data from the 3D stack of blood vessels, with each layer separated by Δz = 1um. Moreover, for each YZ plane in the X direction and each XZ plane in the

Y direction, maximum intensity pixels were saved, and inclination in both direction (α and β, respectively) were calculated based on trend line R2 of the graph (Fig. S3d). Finally, the angle Φ of the blood vessel was determined by the formula related to α and β:

$$\tan^2\Phi = \frac{\tan^2\alpha * \tan^2\beta}{\tan^2\alpha + \tan^2\beta} \quad \text{(S1)}$$

, where the value Φ was always positive, α and β were the angles in the YZ plane (X direction) and XZ plane (Y direction) in space.

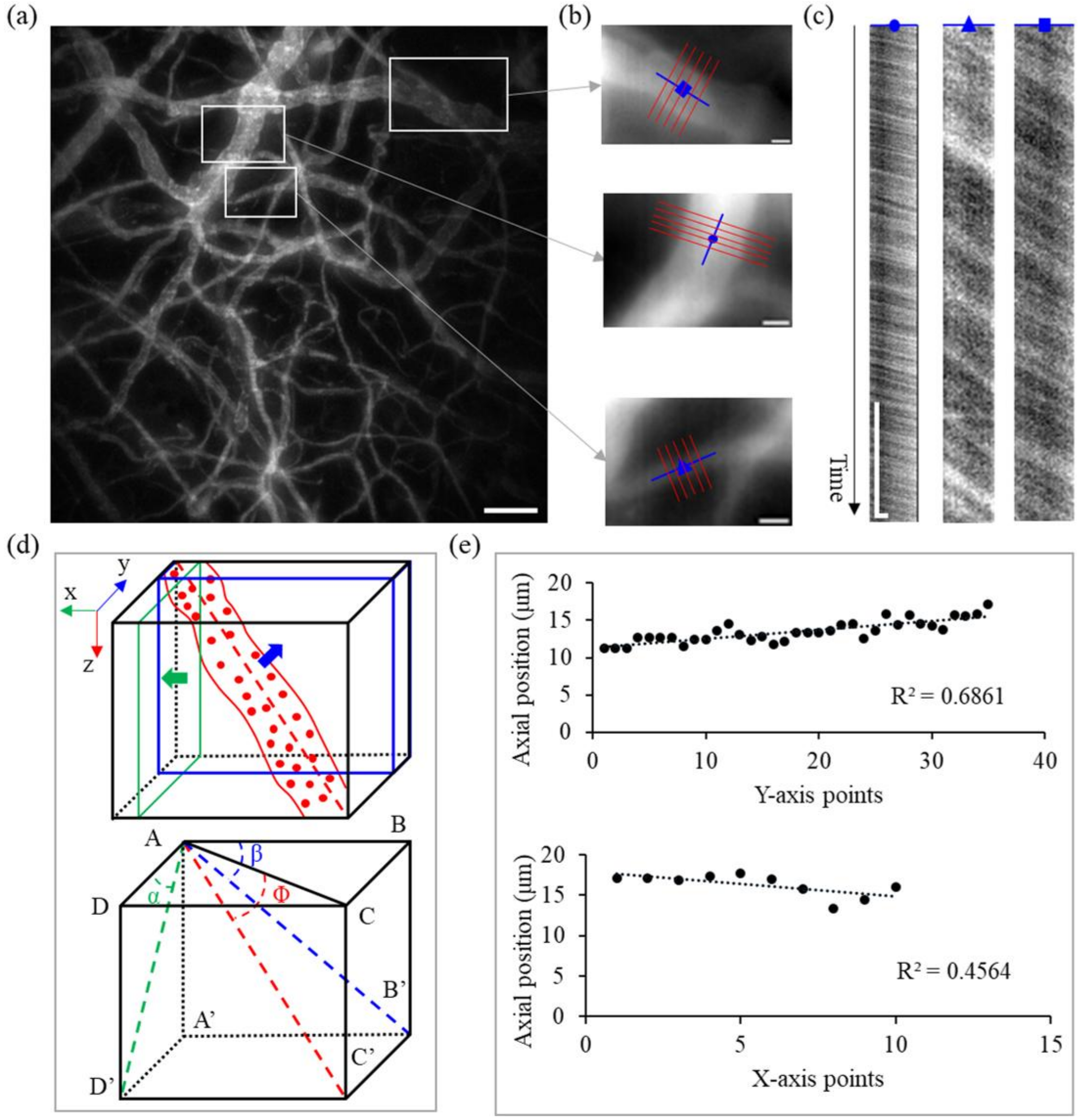

Fig. S3 The quantitative analysis of blood vessel properties: (a) The regions of interest of analyzed vessels. (b) The lines for vessel analysis: blue for measuring velocity and red for measuring vessel diameter. (c) The kymograph and kymograph angle θ analyzed by time. (d) The maximum intensity tracing by pixel in the X and Y direction for calculating the angle Φ. (e) The tilting by X and Y by pixels. Scale bars: 50 μm in (a); 10 μm in (b); 0.5 second and 10 μm in (c).

4. Volumetric imaging with TAG lens

To highlight the effectiveness of TAG lens in volumetric imaging, two images of 1μm fluorescent beads (G0100, Thermo Scientific) sample captured at the same location with the TAG lens on and off were compared (Fig. S4a-b). The TAG lens was activated at 70 % amplitude with a camera speed of 50 fps. Turning off the TAG lens restores the elongated focus, causing the image to be out of focus except for the beads present in the focal plane, which disappear in some cases. This result indicates that TAG-based RASAD enables the acquisition of a projection image covering extended depths with a single scan.

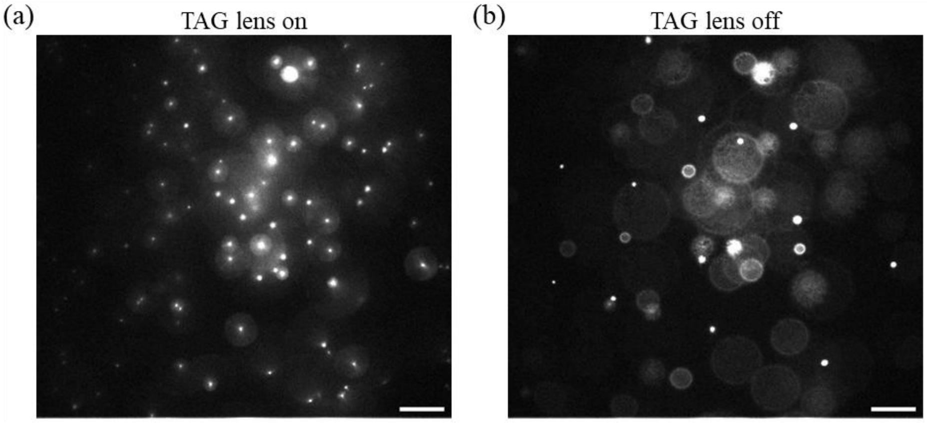

Fig. S4 Volumetric imaging capability of TAG based – RASAD L-SCM: (a) Volumetric image of 1 um fluorescent beads sample with the TAG lens on. (b) Corresponding image with the TAG lens off under the same condition as (a). Scale bar: 50 μm.
